# BCL2L11 Induction Mediates Sensitivity to Src and MEK1/2 Inhibition in Thyroid Cancer

**DOI:** 10.3390/cancers15020378

**Published:** 2023-01-06

**Authors:** Madison M. Rose, Veronica L. Espinoza, Katelyn J. Hoff, Laura A. Pike, Vibha Sharma, Marie-Claude Hofmann, Aik Choon Tan, Nikita Pozdeyev, Rebecca E. Schweppe

**Affiliations:** 1 Division of Endocrinology, Metabolism, and Diabetes, School of Medicine, University of Colorado Anschutz Medical Campus, Mail Stop 7103, Aurora, CO 80045, USA; 2 Department of Cell and Developmental Biology, School of Medicine, University of Colorado Anschutz Medical Campus, Aurora, CO 80045, USA; 3 Department of Pharmaceutical Sciences, Skaggs School of Pharmacy and Pharmaceutical Sciences, University of Colorado Anschutz Medical Campus, Aurora, CO 80045, USA; 4 Department of Endocrine Neoplasia & Hormonal Disorders, Division of Internal Medicine, The University of Texas MD Anderson Cancer Center, Houston, TX 77030, USA; 5 Department of Oncological Sciences, Huntsman Cancer Institute, The University of Utah, Salt Lake City, UT 84112, USA; 6 Division of Bioinformatics and Personalized Medicine, Department Medicine, University of Colorado Anschutz Medical Campus, Aurora, CO 80045, USA; 7 University of Colorado Cancer Center, University of Colorado Anschutz Medical Campus, Aurora, CO 80045, USA

**Keywords:** dasatinib, trametinib, targeted therapy, combination, BIM, thyroid cancer, Src, MAPK

## Abstract

**Simple Summary:**

Thyroid cancer is the most common endocrine malignancy. Patients with advanced thyroid cancers have poor survival rates, largely because of limited therapeutic options to combat their aggressive nature, creating a compelling need to identify novel therapeutic targets. We and others have previously shown that Src is a clinically relevant target in thyroid cancer and that combined inhibition of Src and the MAP kinase pathway results in enhanced anti-tumor responses. The goals of this study were to identify the mechanism(s) mediating these anti-tumor effects and identify additional potential biomarkers of response to improve therapies for patients with advanced thyroid cancer.

**Abstract:**

Patients with advanced thyroid cancer, including advanced papillary thyroid cancer and anaplastic thyroid cancer (ATC), have low survival rates because of the lack of efficient therapies available that can combat their aggressiveness. A total of 90% of thyroid cancers have identifiable driver mutations, which often are components of the MAPK pathway, including *BRAF*, *RAS*, and *RET*-fusions. In addition, Src is a non-receptor tyrosine kinase that is overexpressed and activated in thyroid cancer, which we and others have shown is a clinically relevant target. We have previously demonstrated that combined inhibition of Src with dasatinib and the MAPK pathway with trametinib synergistically inhibits growth and induces apoptosis in *BRAF-* and *RAS*-mutant thyroid cancer cells. Herein, we identified the pro-apoptotic protein BCL2L11 (BIM) as being a key mediator of sensitivity in response to combined dasatinib and trametinib treatment. Specifically, cells that are sensitive to combined dasatinib and trametinib treatment have inhibition of FAK/Src, MEK/ERK, and AKT, resulting in the dramatic upregulation of BIM, while cells that are resistant lack inhibition of AKT and have a dampened induction of BIM. Inhibition of AKT directly sensitizes resistant cells to combined dasatinib and trametinib but will not be clinically feasible. Importantly, targeting BCL-XL with the BH3-mimeitc ABT-263 is sufficient to overcome lack of BIM induction and sensitize resistant cells to combined dasatinib and trametinib treatment. This study provides evidence that combined Src and MEK1/2 inhibition is a promising therapeutic option for patients with advanced thyroid cancer and identifies BIM induction as a potential biomarker of response.

## 1. Introduction

Patients with advanced thyroid cancer, which include advanced papillary thyroid cancer and anaplastic thyroid cancer (ATC), have low survival rates due to their aggressive nature and the lack of effective therapies [1]. The improved understanding of the molecular and genetic drivers of thyroid cancers has led to improved therapeutic options for these patients. Driver mutations are identifiable in >90% of all thyroid cancers, with the most common driver mutations in advanced thyroid cancer patients being mitogen-activated protein kinase, herein referred to as MAPK, pathway mutations in either *BRAF* or *RAS* [2]. The high prevalence of these mutations has led to the clinical development and use of MAPK pathway inhibitors; however, until recently, these inhibitors have had limited success in thyroid cancer. The BRAF inhibitor dabrafenib in combination with the mitogen-activated protein kinase kinase, herein referred to as MEK1/2, inhibitor trametinib has doubled the median overall survival of ATC patients from <6 months to 12 months and is an example of how monumental targeted therapies can be for increasing patient survival [3]. However, while some therapies show initial success, resistance becomes inevitable (acquired resistance), and a significant subset of patients exhibit upfront (intrinsic) resistance. Unlike mutated BRAF, which can be inhibited by several drugs, there is only one FDA-approved therapy that directly targets mutated RAS. Sotorasitib, a KRAS G12C inhibitor, is approved only for non-small cell lung cancer patients, leaving other *RAS* mutant patients limited therapies that have modest efficacy, and creating a compelling need to identify new therapeutic strategies [4].

Src family kinase members, herein referred to as Src, have been shown to be overexpressed and activated in a variety of cancers including breast, colon, lung, and head and neck cancers and contribute to tumorigenic properties, including invasion, metastasis, and a worse survival [5,6,7]. In thyroid cancer, we and others have shown that Src is both overexpressed and activated and that Src inhibition blocks growth, invasion, and metastasis [8,9,10,11,12]. However, like other targeted therapies, it is clear that Src will need to be inhibited in combination with other targets to be most effective. Consistent with this, a phase II clinical trial in ERBB2 (HER2)-positive metastatic breast cancer combined dasatinib with the recombinant antibody targeting HER2 trastuzumab and the chemotherapy drug paclitaxel and had an overall response rate of almost 80%, providing evidence that targeting Src can be clinically beneficial [13]. Accordingly, our previous studies have demonstrated that, in *BRAF-* and *RAS*-mutant thyroid cancer cells, combined Src and MEK1/2 inhibition synergistically inhibits growth in vitro and in vivo through the induction of apoptosis; however, these effects were not achieved in *PIK3CA*-mutant thyroid cancer cells [11,12].

In the present study, we elucidated the mechanism(s) by which inhibition of the Src and the MAPK pathways exert anti-tumor effects, specifically through growth and apoptosis, and identified therapeutic strategies to enhance this effect. Specifically, we found that sensitivity to dual Src and MEK1/2 inhibition was dependent on inhibition of FAK/Src, MEK/ERK, and AKT and the upregulation of the pro-apoptotic protein BCL2L11, herein referred to as BIM. Notably, resistant cells lacked AKT inhibition and the induction of BIM was blunted. We show that an mRNA ratio of ~1.2 for *MCL1:BCL2L1* predicts sensitivity to a BH3 mimetic targeting BCL2L1 (herein referred to as BCL-XL) and that targeting BCL-XL can compensate for the lack of BIM induction in resistant cells. Together, these data indicate that BIM is a convergent point of the Src and MAPK pathways for the regulation of growth and apoptosis and provide a new therapeutic strategy and potential biomarker of response to target these pathways more effectively.

## 2. Materials and Methods

### 2.1. Reagents

Dasatinib (BMS-354825) and trametinib (GSK-1120212) were purchased from LC Laboratories (Woburn, MA, USA); MK2206 and ABT-263 were purchased from Selleckchem (Houston, TX, USA). A-1210477 was purchased from Sigma Aldrich (St. Louis, MO, USA). All drugs were dissolved in dimethyl sulfoxide at 10 mM stocks.

### 2.2. Cell Culture

All cell lines listed in Table S1 are of human thyroid cancer origin, grown in their recommended media, and maintained at 37 °C in 5% CO_2_. All cell lines were validated using short tandem repeat (STR) profiling using the Applied Biosystems Identifier Kit (#4322288, Waltham, MA, USA) or GlobalFiler PCR Amplification Kit (#4476135, Warrington, UK) in the Barbara Davis Center BioResources Core Facility, Molecular Biology Unit, at the University of Colorado. Cells were tested for *Mycoplasma* contamination using the Lonza Mycoalert system. Cell lines were passed no more than 30 times after thawing.

### 2.3. Cell Viability Assay

Cell Titer Glo: Cells (1000 cells/well) were plated in opaque-walled 96-well plates, in 100 μL of their respective media. Cells were treated with 8 concentrations of dasatinib (19–1250 nM) with or without trametinib (10 or 100 nM) for 72 h, and cell viability was measured using CellTiterGlo 2.0 assay (Promega, Madison, WI, USA) following the manufacturer’s protocol. Luminescence was measured using a BioTek Synergy H1 Plate Reader (Winooski, VT, USA); viability was calculated in Excel, and IC_50_ values were calculated in GraphPad Prism 9 (San Diego, CA, USA) using nonlinear regression.

Sulforohodamine B (SRB) Assay: Cells (1100–2200/well) were plated in triplicate in 96-well plates in 100 μL of their respective media. Cells were treated with 8 concentrations of dasatinib (19–1250 nM) with or without trametinib (10 or 100 nM), and cell growth was measured by SRB assay (Sigma) after 3 days of drug treatment, as previously described [14].

### 2.4. Micorarray Gene Expression Profiling and RNA Sequencing

Transcriptome-wide Affymetrix gene expression data for the thyroid cancer cell line panel was analyzed from our previously published data set [15]. For the RNA sequencing experiment, cells were plated at a density range of 0.6–1.2 × 10^6^ depending on cell line in 10 cm plates and allowed to adhere overnight. The next day, cells were treated with vehicle, 50 nM dasatinib, 100 nM trametinib, or the combination and harvested 48 h later. RNA was extracted using Qiagen RNeasy Plus Kit (74034, Hilden, Germany) and quantified using Take 3 plate on the BioTek Hybrid Synergy1 Plate Reader. The Genomics and Microarray Shared Resource (University of Colorado Cancer Center) performed mRNA sequencing in the NovaSEQ 6000 sequencing platform (paired end 150 cycles). To determine enriched pathways between sensitive and resistant cell lines, from the Affymetrix dataset, the 3 most sensitive and 3 most resistant cells based off IC_50_ values were compared. Similarly, from the RNA sequencing experiment gene expression, dataset reads per kilobase per million (RPKM) from 3 sensitive and 2 resistant cell lines were estimated and compared based off IC_50_ values. Both datasets were analyzed using Gene Set Enrichment Analysis (GSEA). The pathways from the Hallmark Genes were used as the gene set, and permutations were set to 1000.

### 2.5. Reverse Phase Protein Array

A Reverse Phase Protein Array (RPPA) was performed at the Functional Proteomics RPPA Core Facility at MD Anderson, as previously described [16]. Briefly, cells were seeded at a density of 0.6–1.2 × 10^6^ cells in a 10 cm plate with RPMI supplemented with 5% FBS and allowed to adhere overnight. Lysates were then collected, diluted in five 2-fold serial dilutions, and arrayed on nitrocellulose-coated slides using an Aushon Biosystems 2470 arrayer (Billerica, MA, USA). Each slide was then probed with a primary antibody, followed by a biotin conjugated secondary antibody. Protein concentrations were then normalized for protein loading and corrected for by median centering across samples and median centering across antibodies. Protein analysis was then performed at the MD Anderson Functional Proteomics RPPA Core Facility. Apoptosis proteins on the RPPA were isolated based on the apoptosis genes listed on MD Anderson Pathway Browser (ID 2991). Multiple unpaired *t*-tests correcting for multiple comparisons between vehicle treated and combination treated cells from the RPPA were used to calculate statistical differences. Ordinary one-way ANOVA comparing the mean of each column with the mean of every other column with Tukey’s correction for multiple comparisons was performed on BIM RPPA data using Graph Pad Prism 9.

### 2.6. Immunoblotting

Cells were collected in CHAPS lysis buffer (containing 10 mmol/L CHAPS, 50 mmol/L Tris (pH 8.0) 150 mmol/L NaCl, and 2 mmol/L EDTA or RIPA lysis buffer (containing 150 mmol/L NaCl, 50 mmol/L Tris (pH 8.0) 1% Nonidet P-40 substitute, 0.5% sodium deoxycholate, and 0.1% SDS) with 1× protease/phosphatase inhibitor cocktail (Thermo). Protein (20 μg) was separated using 4–20% PAGE-SDS gels and transferred to Immobilon-P membranes (Millipore). Membranes were incubated overnight at 4 °C with the indicated antibodies. Antibodies were purchased from Cell Signaling: BIM (2933), Beta Actin (3700), pAKT S473 (4060), Total AKT (2920), pSrc Y416 (6943), Total Src (2109), BD Biosciences: Total FAK (610087), Invitrogen: pFAK Y861 (44-626G), and CalbioChem: α-tubulin (CP06). Blots were imaged using the Odyssey Clx Imager (Li-Cor, Lincoln, NE, USA) and quantified using Image Studio (Li-Cor). RRID for antibodies can be found in Table S2, and the original blots are shown in Figure S6.

### 2.7. siRNA Experiments

The small interfering RNA (siRNA) constructs were obtained as the siGENOME SMARTpool reagents (Dharmacon), the siGENOME SMARTpool BIM (M-004383-02-0050), and the nontargeting siRNA control, siRNA Pool #1 (D-001206-13-50). For cell viability assays, cells were plated at a density of 3000–3500 cells/well in 96-well plate for a reverse transfection at a final concentration of 50 nM of siRNA in Opti-MEM medium (Invitrogen) using lipofectamine RNAiMAX reagent (Invitrogen). For Western blots, cells were plated at a density of 1.5 × 10^6^ cells in a 10 cm plate for reverse transfection at a final concentration of 50 nM of siRNA.

### 2.8. Doxycycline Inducible pTREX Expression Vectors

Empty vector and BIM vector plasmids were kindly provided by Dr. Anthony Faber (Department of Oral and Craniofacial Molecular Biology, Virginia Commonwealth Philips Institute). Lentiviral transduction of empty vector and BIM constructs were prepared as previously described [12]. CUTC60 and T238 cells (low BIM expressing cells) were transduced with empty vector or the BIM vector and selected in 2.5 μg/μL and 3 μg/μL puromycin in RPMI supplemented with 5% FBS. Following selection, cells were titrated with doxycycline (DOX) to find a concentration of DOX that induced BIM to levels similar to levels found in high BIM-expressing cells. For all subsequent experiments, either DOX or control was added upon plating of cells, and 24 h later, cells were treated with vehicle, the doses of dasatinib and trametinib as indicated in the figure lengends, either as single agents or in combination.

### 2.9. Myristoylated AKT

pBabe Puro-Myr-Flag-AKT1 was a gift from William Hahn (Addgene plasmid #15294) [17]. pBabe empty vector was a gift from Jay Morgenstern, Bob Weinberg, and Hartmut Land (Addgene plasmid #1764) [18]. Retroviral particles were generated, and *BRAF*-mutant 8505C was transduced with myr-AKT1 virus or pBABE empty vector and selected in 2 μg/μL of puromycin in RPMI supplemented with 5% FBS as previously described [12].

### 2.10. Apoptosis Assays

Annexin V and Propidium Iodide Staining: Apoptosis was measured using Annexin V and propidium iodide staining. Cells were plated at a density of 1.5 × 10^6^ cells in a 10 cm dishes. BIM knockdown cells were plated for reverse transfection at a final concentration of 50 nM of siRNA. The next day cells were treated with 50 nM dasatinib and 100 nM trametinib for 24 h in RPMI media supplemented with 5% FBS. Following treatment, cells from the supernatant were collected, and adherent cells were detached with 3 mM EDTA in PBS. Cells were then stained with Annexin V FITC and propidium iodide according to an eBioscience^TM^ Annexin V Apoptosis Detection Kit FITC. Cells were analyzed using the ZE5^TM^ Cell Analyzer University of Colorado Cancer Center Flow Cytometry Shared Resource.

Cleaved Caspase 3/7: Cells (1000 cells/well) were plated in black-walled 96-well plates in 100 μL of RPMI supplemented with 5% FBS. Cells were treated with 50 nM dasatinib, 100 nM trametinib, alone or in combination, and Caspase-3/7 Dye (Sartorius Cat. 4440, Bohemia, NY, USA) for apoptosis was added for a final concentration of 5 μM. Apoptosis was measured using an Agilent Cytation 5 Cell Imaging Multimode Reader for 72 h, with images taken in 4 h increments. Data reduction steps were generated, and the induction of cleaved caspase 3/7 was measured as green object counts for each timepoint. Timepoints were graphed in GraphPad Prism 9, and the area under the curve was calculated for vehicle, dasatinib, trametinib, or the combination. Data was then normalized to vehicle and graphed.

### 2.11. Statistical Analysis

All experiments were performed in biological and technical triplicates and analyzed for statistical significance using the GraphPad Prism (Version 9.4.1). One-way ANOVA was used to compare the means of three or more independent groups. Error bars represent the SEM, unless otherwise noted in their respective figure legends.

## 3. Results

### 3.1. Combined Treatment with Dasatinib and Trametinib Identifies BIM as a Mediator of Sensitivity

The growth inhibitory effects of the Src inhibitor, dasatinib, in combination with the MEK1/2 inhibitor trametinib were analyzed in 23 thyroid cancer cell lines expressing clinically relevant mutations (*BRAF*, *RAS*, *RET/PTC1*). Dasatinib and trametinib were chosen for these studies based on their stage in clinical development in thyroid and other tumor types [19,20,21]. Trametinib is a potent and highly selective inhibitor of MEK1/2 [22].While dasatinib is a potent inhibitor of Src; similar to other Src inhibitors, it is also a multi-tyrosine kinase inhibitor [23]. Thus, we have taken several approaches to rigorously test the specific role of Src in thyroid cancer, including genetic approaches (shRNA and expression of a drug-resistant c-Src gatekeeper mutation) and treatment with dasatinib doses < 100 nM to control for off-target effects, along with treatment with two distinct Src inhibitors, which show similar anti-tumor responses [10,11,12,24]. Together, at this time, our data support the use of dasatinib as the best clinically relevant Src inhibitor for these studies.

**Figure 1:**
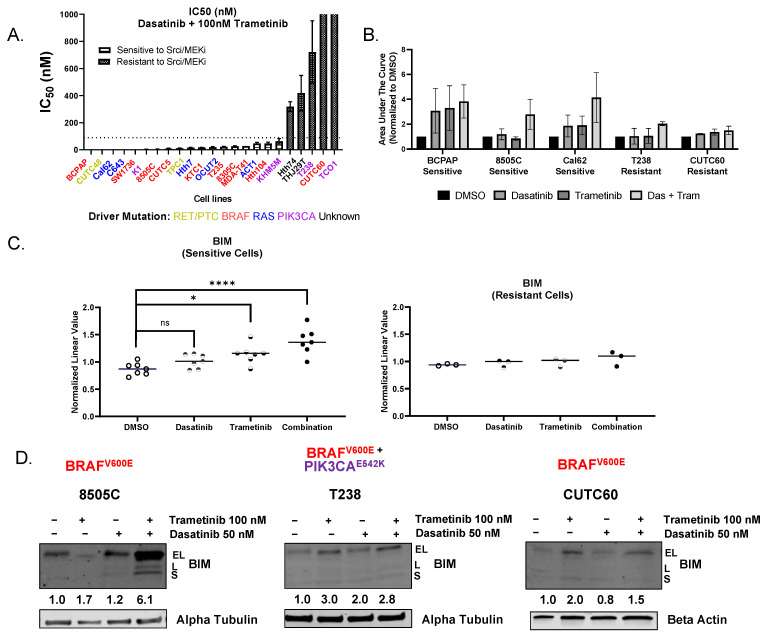
Dasatinib and trametinib treatment inhibits cell growth, induces apoptosis, and increases BIM expression. (**A**) Absolute quantification of the IC_50_ value for dasatinib with 100 nM trametinib in 23 thyroid cancer cell lines. Viability curves were measured across the cell lines using CellTiter-Glo Assay (Promega) or Sulforohodamine B Assay (SRB). Dashed line indicates 90 nM IC_50_ cutoff used to determine sensitivity and resistance to dasatinib and trametinib. Results shown are mean ± SEM. (**B**) Cleaved caspase 3/7 activity over the course of 3 days was measured in 3 sensitive cells (BCPAP, 8505C, and Cal62) and 2 resistant cells (T238 and CUTC60), treated alone or in combination with dasatinib and trametinib at 50 nM and 100 nM concentrations, respectively, and graphed as area under the curve. Results shown are mean ± SEM. (**C**) BIM expression obtained from RPPA data in sensitive and resistant cell lines. “ns” indicates not significant, **** *p* < 0.0001, * *p* < 0.01 (**D**) Immunoblot analysis of 8505C, T238, CUTC60 cells treated with indicated concentrations of dasatinib, trametinib, or the combination for 24 h and analyzed by Western blot for expression of BIM isoforms. EL = extra long, L = long, S = short. Alpha tubulin or beta actin were used as a loading control. Numbers below represent densitometric analysis normalized to loading control, followed by DMSO-treated cells. The uncropped blots are shown in Figure S6.

To define cell lines that are responsive to combined Src and MEK1/2 inhibition, we chose 90 nM as the IC_50_ cutoff for dasatinib based on the selectivity of dasatinib and the peak/plasma concentration in chronic myelogenous leukemia patients [19]. Cells were treated with increasing doses of dasatinib (0 to 1250 nM) in the presence of 10 or 100 nM trametinib, and growth inhibition was measured using CellTiter Glo or Sulforhodamine B (SRB) assays (Figure 1A, Table S3). IC_50_ values for dasatinib +/− trametinib were calculated, and Figure 1A shows that 18 out of 23 thyroid cancer cell lines were sensitive to dasatinib in combination with 100 nM trametinib (Figure 1A, Table 1). Similar results were observed with dasatinib in combination with 10 nM trametinib, with 14 out of 23 thyroid cell lines being sensitive (Table S3).

We next asked whether sensitivity to combined Src and MEK1/2 inhibition was associated with driver oncogene mutations, and, as shown in Figure 1A, no correlation between *BRAF*, *RAS*, *RET/PTC1*, or *PIK3CA* was observed. To determine whether other cancer-related genes may correlate with Src and MEK1/2 inhibitor sensitivity, we analyzed our previously published mutational analysis of thyroid cancer cell lines and did not observe a correlation with other oncogenic mutations, including *TERT* promoter mutations or dasatinib off-targets [15]. Overall, these data are consistent with our previous study showing that c-Src is the top protein correlated with dasatinib sensitivity rather than driver mutations or off-targets [12].

To determine the contribution of apoptosis, we next measured cleaved caspase 3/7 as a readout of apoptosis and quantitated apoptotic responses by the calculated area under the curve (AUC). For these studies, we used representative sensitive cell lines expressing *BRAF^V600E^* mutations (BCPAP, 8505C) or *KRAS^G12R^* mutation (Cal62) and resistant cell lines expressing *BRAF^V600E^ PIK3CA^E542K^* mutations (T238) or *BRAF^V600E^* (CUTC60). Consistent with our previous data, the representative sensitive cell lines demonstrated an enhanced induction of cleaved caspase 3/7 in response to dasatinib and trametinib treatment compared to vehicle (3-fold to 4-fold increase in apoptosis; Figure 1B) [12]. In contrast, the representative resistant cell lines (T238, CUTC60) exhibited minimal induction of cleaved caspase 3/7 in response to combined dasatinib and trametinib treatment (Figure 1B). Together these results indicate that combined Src and MEK1/2 inhibition blocks growth and induces apoptosis in thyroid cancer cells that cannot be predicted by oncogene mutations.

We next took unbiased proteomic and transcriptomic approaches to evaluate proteins and/or gene regulatory networks with the potential to regulate apoptosis and serve as potential therapeutic targets and/or biomarkers of response. We utilized two different gene expression datasets along with reverse phase protein array (RPPA) on 7 representative sensitive and 3 representative resistant cell lines (Table 1). We performed gene set enrichment analysis (GSEA) using the hallmarks gene set on our two gene expression data sets, which revealed enrichment of the hallmark signature for apoptosis in the sensitive cells in both data sets (at baseline) (*p* < 0.001, *p* = 0.04 Figure S1A,B). To directly evaluate changes at the protein level, we used RPPA, which includes over 425 antibodies that recognize activated pathways through phosphorylated proteins, including MAPK, PI3K/AKT, and FAK/Src, as well regulators of cell mechanisms like autophagy, apoptosis, cell cycle, and DNA repair. Cells were treated with vehicle or the indicated doses of dasatinib, trametinib, or the combination for 24 h. As expected, treatment with dasatinib or trametinib resulted in the inhibition of the FAK/Src and MAPK pathways, respectively (Figure S1E). Our analysis of apoptosis-related proteins showed that three proteins were significantly increased in sensitive cells treated with the combination, one of which was the BH3-only protein BIM (Figure S1C).

In the sensitive cells (8505C, BCPAP, Cal62, SW1736, C643, MDA-T41, K1), BIM protein expression was significantly increased with single-agent trametinib treatment (*p* < 0.05) and further enhanced with the combination treatment compared to vehicle (*p* < 0.005, Figure 1C). In the resistant cells (T238, CUTC60, TCO1), BIM protein expression remained unchanged regardless of treatment (Figure 1C). Interestingly the mean expression of BIM between the sensitive and resistant cells differs significantly in the combination treated cells (*p* = 0.02, Figure S1D). Induction of BIM was validated in representative sensitive (8505C) and resistant (T238, CUTC60) cells. Specifically, in 8505C cells we observed a 6-fold induction of BIM in combination treated cells compared to vehicle (Figure 1D). In the resistant T238 and CUTC60 cells, we observed minimal changes of BIM in response to combined dasatinib and trametinib treatment- compared to vehicle (Figure 1D). These data indicate that BIM is a potential biomarker of response to combined Src and MEK1/2 inhibition.

### 3.2. Induction of BIM Is Required for Growth Inhibition and Apoptosis Induction by Combined Dasatinib and Trametinib

**Figure 2:**
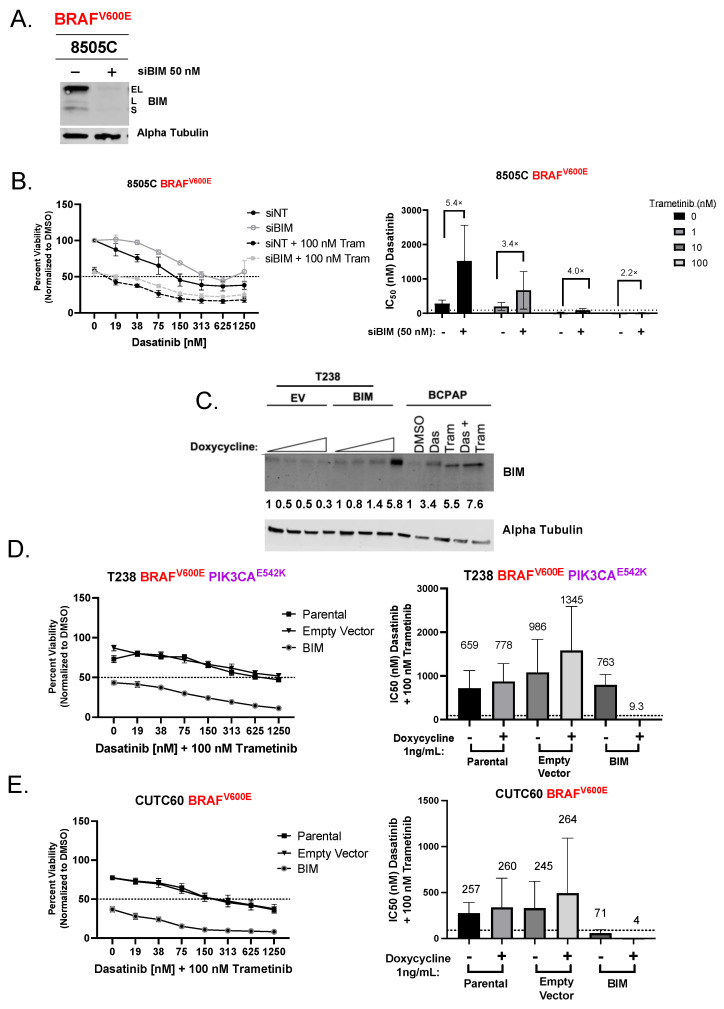
Growth inhibition by dasatinib and trametinib is dependent on BIM. (**A**) 8505C cells reverse transfected with nontargeting siRNA (siNT) or siRNA targeting BIM (siBIM). Cells were treated with 50 nM dasatinib and 100 nM trametinib for 24 h to induce BIM. (**B**) CellTiter Glo assays for viability were performed on siNT or siBIM cells. Cells were treated with indicated concentration of dasatinib with or without 100 nM trametinib for 72 h. Dashed line indicates 50% viability. Data was normalized to DMSO-treated control set to 100%. Results shown are mean ± SEM. IC_50_ values were calculated, and fold change is listed above the bars. (**C**) Immunoblot analysis of T238 cells overexpressing an empty vector (EV) or BIM plasmid. Cells were treated with 0, 0.5, 0.75, or 1 ng/mL of doxycycline upon plating. BCPAP cells served as a positive control for BIM induction and were treated with DMSO, 50 nM dasatinib, 100 nM trametinib, or the combination. Alpha tubulin was used as a loading control. Numbers below represent densitometric analysis normalized to loading control and 0 doxycycline- or DMSO-treated cells. (**D**,**E**) CellTiter Glo assay for viability was performed on parental, empty vector, or BIM-expressing cells. Cells were treated with indicated concentrations of dasatinib with or without 100 nM trametinib for 72 h. Data was normalized to DMSO-treated control set to 100%. Results shown are mean ± SEM. IC_50_ values were calculated and listed above the bars. The uncropped blots are shown in Figure S6.

We next assessed the functional role of BIM by knocking down BIM in the sensitive 8505C cells using a pool of four siRNAs targeting either BIM (siBIM) or a nontargeting control (siNT). Using this approach, we achieved >90% knockdown of BIM 24 h post-transfection (Figure 2A). To assess the role of BIM in growth inhibition, we treated siNT or siBIM cells with dasatinib (0 to 1250 nM) alone or in combination with trametinib (0 to 100 nM), and cell viability was measured using Cell Titer Glo 3 days post-treatment. Figure 2B shows that knockdown of BIM increased the IC_50_ of dasatinib alone by 5.4-fold compared to the siNT cells (siNT = 280 nM siBIM = 1520 nM), and the IC_50_ of dasatinib and 100 nM trametinib was increased by >2-fold (siNT = 8 nM siBIM = 18 nM). Accordingly, knockdown of BIM moderately decreased total apoptosis in the 8505C cells from 25 ± 3.5%, to 17 ± 0.3% (*p* = 0.10, Figure S2). These data indicate that BIM is a key mediator of growth and apoptosis in response to Src and MEK1/2 inhibition.

Based on our data, we hypothesized that there is a threshold level of BIM induction required for sensitivity to combined Src and MEK1/2 inhibition. To test this hypothesis, we overexpressed BIM, using a doxycycline-inducible plasmid, in two resistant cell lines to levels similar to those in sensitive cells (T238 Figure 2C), and cell viability was measured as described above. In the T238 cells, induction of BIM led to an 82-fold decrease in the IC_50_ of dasatinib in the presence of 100 nM trametinib (-BIM = 763 nM, + BIM = 9.3 nM Figure 2D and Figure S3A). Similarly, in the CUTC60 cells, induction of BIM led to an 18-fold decrease in the IC_50_ of dasatinib plus 100 nM trametinib (-BIM = 71 nM, + BIM = 4 nM) (Figure 2E and Figure S3A). Furthermore, T238 and CUTC60 BIM-overexpressing cells had a greater induction of cleaved caspase 3/7 (0.9- to 2-fold, 1.5- to 4.7-fold) when treated with combined dasatinib and trametinib compared to the empty-vector-expressing cells (*p* ≤ 0.01, *p* ≤ 0.0001 Figure S3B). Together, these data support our hypothesis that a threshold level of BIM is required to mediate growth inhibition and induction of apoptosis in response to combined Src and MEK1/2 inhibition.

### 3.3. Inhibition of AKT Is Necessary for Sensitivity to Combined Dasatinib and Trametinib

**Figure 3:**
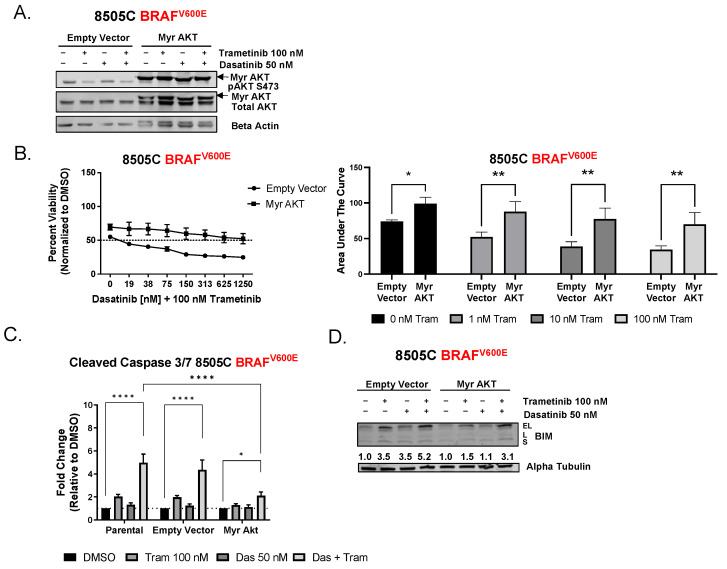
Constitutively active AKT increases resistance to combined dasatinib and trametinib. (**A**) 8505C empty vector or Myr AKT cells were treated with indicated concentrations of dasatinib, trametinib, or the combination for 24 h and analyzed by Western blot for confirmation of pAKT. (**B**) CellTiter Glo assay for viability was performed on 8505C cells transfected with empty vector or myristoylated AKT (Myr AKT). Cells were treated with indicated doses of dasatinib and with 100 nM trametinib for 72 h. Area under the curve was calculated, and the results shown are mean ± SEM 2-way ANOVA * *p* < 0.05 ** *p* < 0.005. (**C**) Cleaved caspase 3/7 activity was measured after parental, empty vector, and Myr AKT cells were treated with DMSO, 100 nM trametinib, 50 nM dasatinib, or the combination for 24 h. Results shown are mean ± SEM. 2-way ANOVA test **** *p* < 0.003 * *p* < 0.03 (**D**) 8505C empty vector or Myr AKT cells were treated with indicated concentrations of dasatinib, trametinib, or the combination for 24 h and analyzed by Western blot for expression of BIM. Alpha tubulin was used as a loading control. Numbers below represent densitometric analysis normalized to loading control, followed by DMSO-treated cells. The uncropped blots are shown in Figure S6.

We previously published that increased PI3K signaling correlates with resistance to Src inhibition and that cells resistant to combined Src and MEK1/2 inhibition had sustained levels of phospho-AKT [12]. Therefore, we hypothesized that activation of the AKT pathway is a mechanism of resistance to combined Src and MEK1/2 inhibition. To test this hypothesis, we transduced an empty vector (EV) or constitutively active AKT (Myr AKT) into the 8505C (sensitive to combination) cells and measured cell viability, as described above (Figure 3A). Figure 3B shows that expression of Myr AKT in the 8505C cells shifted the IC_50_ from 8 nM to >1250 nM, with a corresponding 2-fold increase in AUC compared to the empty vector cells (*p* = 0.004, Figure 3B and Figure S4). Accordingly, the 8505C Myr AKT-expressing cells exhibited only a 2-fold induction of cleaved caspase (*p* ≤ 0.03 Figure 3C) compared to a 4-fold induction of cleaved caspase in the empty-vector-expressing cells (*p* < 0.0001). Consistent with a role for BIM in this response, we show that 8505C Myr AKT expressing cells exhibit a blunted induction of BIM compared to the empty vector cells (Figure 3D). Together, these results indicate that AKT blocks the induction of BIM to promote survival and prevent apoptosis in response to combined Src and MEK1/2 inhibition.

**Figure 4:**
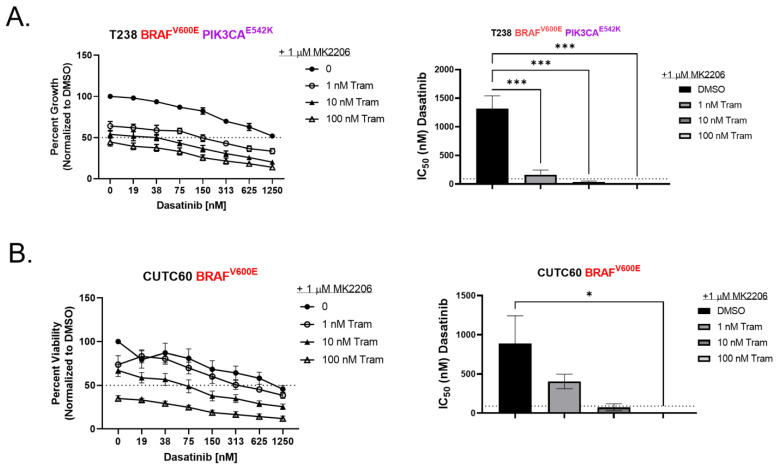
The addition of the AKT inhibitor MK2206 increases the efficacy of dasatinib and trametinib. (**A**,**B**) CellTiter Glo assay for viability was performed on cells treated with indicated doses of dasatinib, trametinib, and 1 μM of MK2206 for 72 h. Dashed line indicates 50% viability. Data were normalized to DMSO-treated control set to 100%. Results shown are mean ± SEM. IC_50_ values were calculated in GraphPad Prism ordinary one-way ANOVA test *** *p* < 0.006 * *p* < 0.06.

Based on these results, we further hypothesized that inhibition of AKT in the resistant cells will sensitize cells to combined Src and MEK1/2 inhibition, phenocopying overexpressing BIM. Accordingly, the addition of the AKT inhibitor, MK2206, to the dasatinib and trametinib combination, was sufficient to sensitize T238 (resistant to combination) (IC_50_ = 8.5 nM *p* = 0.0003) (Figure 4A) and CUTC60 (resistant to combination) (IC_50_ = 2.4 nM *p* = 0.04) (Figure 4B) cells to Src and MEK1/2 inhibition.

### 3.4. The RNA Ratio of MCL1:BCL-X_L_ Predicts Sensitivity to the BH3 Mimetic ABT-263

**Figure 5:**
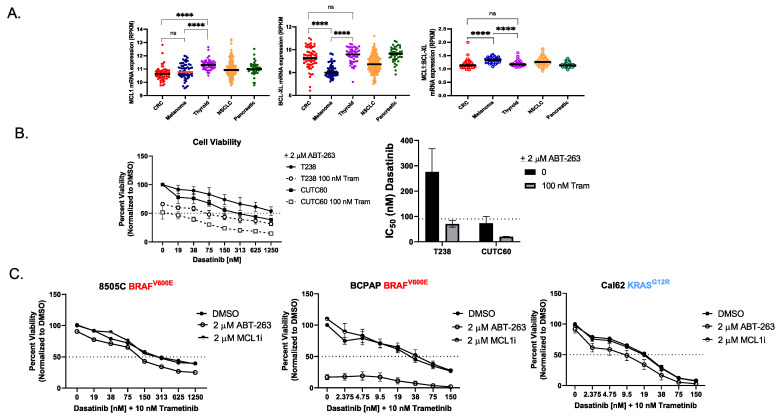
The mRNA ratio of *MCL1:BCL-XL* predicts sensitivity to the pan BCL-2 inhibitor ABT-263. (**A**) mRNA RPKM values for *MCL1* and *BCL-XL* were obtained from the Cancer Cell Line Encyclopedia (CCLE). Ordinary one-way ANOVA test **** *p* < 0.0001, “ns” indicates not significant. (**B**) CellTiter Glo assay for viability was performed on T238 and CUTC60 cells. Cells were treated with indicated doses of dasatinib, 2 μM ABT-263, with or without 100 nM trametinib for 72 h. Data was normalized to DMSO-treated control set to 100%. Dashed line indicates 50% viability. Results shown are mean ± SEM. IC_50_ values for dasatinib with 2 μM ABT-263 and dasatinib with 2 μM ABT-263 and 100 nM trametinib were calculated in GraphPad Prism. 90 nM cutoff for sensitivity is indicated by the dashed line. (**C**) CellTiter Glo assay for viability was performed on 8505C, BCPAP, and Cal62 cells. Cells were treated indicated doses of dasatinib, 2 μM ABT-263 or 2 μM of an MCL1 inhibitor (MCL1i = A-1210477) with 10 nM trametinib for 72 h. Data were normalized to DMSO-treated control set to 100%. Dashed line indicates 50% viability. Results shown are mean ± SEM.

Our data indicate that inhibition of AKT is necessary to sensitize resistant cells to combined Src and MEK1/2 inhibition; however, a triple-combination therapy of an AKT, MAPK, and Src inhibitor would likely be clinically intolerable [25,26,27]. As an alternative approach, we turned to the use of BH3 mimetics as a potential strategy to induce sensitivity to combined Src and MEK1/2 inhibition. To identify the best BH3 mimetic strategy, we analyzed mRNA ratios of *MCL1* and *BCL-XL*, based on a previous study that showed a low ratio of *MCL1:BCL-XL_,_* in colorectal cancer (CRC) correlated with synthetic lethality between ERK pathway inhibitors and pan BCL-2 inhibitors [28]. Using this approach, we found that thyroid cancer cells exhibit a ratio of 1.199 *MCL1:BCL-XL* that most closely aligns with CRC’s ratio of 1.163. Therefore, we chose to use a pan BCL-2 inhibitor, ABT-263, in combination with dasatinib and trametinib (Figure 5A). In the presence of ABT-263, the IC_50_ value of combined dasatinib and trametinib treatment decreased ~4-fold in the T238 cells (IC_50_^Das+Tram^ = 251 nM, IC_50_^Das, Tram, ABT-263^ = 70 nM) and >40-fold in CUTC60 cells (IC_50_^Das+Tram^ = 861 nM, IC_50_^Das, Tram, ABT-263^ = 20 nM) (Figure 1A and Figure 5B). Furthermore, we demonstrate that the addition of ABT-263 enhances the efficacy of combined Src and MEK1/2 inhibition in sensitive cells 8505C, BCPAP, and Cal62 by >2-fold, while the mimetic targeting MCL1 had no efficacy (*p* ≤ 0.0001, Figure 5C). The most notable toxicity of BH3 mimetics is thrombocytopenia, which is time- and dose-dependent [29]. To begin to assess potential sequential treatment options, we provide evidence that 24 h post Src and MEK1/2 inhibition, BIM is induced and remains elevated for at least 7 days post-treatment (Figure S5). This sustained elevation of BIM may present an opportunity to temporally separate the addition of a BH3 mimetic from Src and MEK1/2 inhibitor treatments and reduce overall toxicities. Taken together our data show that the addition of a BH3 mimetic targeting BCL-XL in combination with dasatinib and trametinib is sufficient to overcome intrinsic resistance to Src and MEK1/2 inhibition as well as enhance efficacy of this combination.

## 4. Discussion

There is a compelling need for new therapeutic options for patients with advanced thyroid cancer. Mutations in the MAPK pathway are highly prevalent in thyroid tumors; however, there has been mixed success targeting the MAPK pathway in advanced PTC and ATC patients. We and others have identified the Src and the MAPK pathways as alternative, targetable pathways in thyroid cancer and importantly, represent a potential therapeutic strategy for targeting thyroid cancers with various oncogenic drivers [11,12,30,31]. In the present study, we set out to determine the mechanism(s) by which Src and the MAPK pathways induce growth inhibition through the induction of apoptosis to identify new therapeutic strategies and potential biomarkers of response. Using the Src inhibitor dasatinib with the MEK1/2 inhibitor trametinib in a panel of thyroid cancer cells, we identified BIM as key mediator of the apoptotic response (Figure 1). Specifically, we identified BIM as a functional biomarker of response, in which knockdown of BIM in sensitive cells increases resistance to Src and MEK1/2 inhibition (Figure 2A,B), and the re-introduction of BIM in resistant cells is sufficient to sensitize cells to combined Src and MEK1/2 inhibition (Figure 2C–E).

As Src is rarely mutated in thyroid or other solid tumors, there are currently neither predictive nor post-treatment biomarkers for Src inhibitors, which has hampered the clinical development of Src inhibitors. Herein, we have shown that sensitivity to combined Src and MEK1/2 inhibition does not correlate with oncogenic mutations in thyroid cancer (Figure 1), which is consistent with our previously published data [12]. Instead, our data reveal that cells sensitive to Src and MEK1/2 inhibition are enriched for apoptosis genes at baseline, suggesting these cells are primed for apoptosis. Importantly, this gene enrichment signature could be used clinically to distinguish between patients who would and would not respond to Src and MEK1/2 inhibition. In addition, our study demonstrates that BIM is a potential functional biomarker of response that can be tested in thyroid cancer and other tumors in response to Src and MEK1/2 inhibition, including non-small cell lung, breast, and ovarian cancers, which are dependent on cooperative Src and MAPK signaling [32,33,34]. In support of this, BIM is already used as a predictive biomarker for other inhibitors, including anti-PD-1 therapy, EGFR, HER2, and PI3K inhibitors [35,36,37]. Thus, our studies are of high clinical significance and provide a much-needed biomarker for combined Src and MAPK pathway inhibition.

Our previous study indicated a potential role for PI3K/AKT signaling as a predictive biomarker of intrinsic resistance to single-agent Src inhibition [12]. In this study, we went on to show that cells sensitive to Src and MEK1/2 inhibition exhibited inhibition of three key nodes: Src/FAK, MEK/ERK, and PI3K/AKT (Figure S1E) [12]. Herein, we further show the functional importance of AKT in this response, wherein ectopic expression of a constitutively active AKT blocked growth inhibition and the induction of apoptosis mediated by Src and MEK1/2 inhibition (Figure 3). Consistent with our studies, Anderson et al. also demonstrated synergy between Src and MEK1/2 inhibition through the inhibition of the same key nodes: Src, MEK/ERK, and AKT [38]. Herein, we further link levels of the pro-apoptotic protein, BIM, to the induction of apoptosis by combined Src and MEK1/2 inhibition through the activation of AKT. We show that constitutive activation of AKT blocks the induction of BIM previously observed in response to Src and MEK1/2 inhibition (Figure 3). Together, these data indicate that BIM is a point of convergence for the Src and MAPK pathways. Of interest, phosphorylation of BIM by ERK at Serine 69 mediates the proteasomal degradation of BIM, which could account for the upregulation of BIM and induction of apoptosis in response to MAPK inhibition observed here (Figure 1C,D and Figure 3D) [39]. In addition, AKT is known to regulate BIM through phosphorylation at serine 87. Thus, we speculate that the regulation of BIM by Src is mediated through the ability of Src to regulate AKT (Figure S1E and Figure 3A) [12]. Of further interest, Src has been shown to directly phosphorylate AKT on Tyr-315, which will be of interest to evaluate in future studies [40].

Finally, we demonstrate that resistance to Src and MEK1/2 inhibition can be overcome by the addition of an AKT inhibitor, though a triple-combination therapy will likely be toxic in the clinic based on the toxicities reported in clinical trials combining AKT and MEK1/2 inhibitors [26,27]. As an alternative approach, we propose the use of BH3 mimetics. In addition, we provide evidence that a low mRNA ratio of *MCL1:BCL-XL* is indicative of sensitivity to a BCL-XL mimetic over an MCL1 mimetic; thus the ratio of *MCL1:BCL-XL* could be used clinically as a guide to which BH3 mimetic to use. Compared to melanoma, the success of MAPK-directed therapies in thyroid cancer, with the exception of ATC and colorectal cancers, has been largely limited [41,42,43]. Our study also reveals the ratio of *MCL1:BCL-XL* is similar between thyroid and colorectal cancer, suggesting that colorectal cancer may also benefit from this triple-combination therapy.

Insights from this study and future studies will better guide therapeutic options for patients with advanced thyroid cancer and other tumors dependent on Src and MAPK signaling. Our studies have focused on the anti-tumor effects of Src and MEK1/2 inhibition on thyroid cancer cells using in vitro approaches. Thus, in future studies, it will be of interest to determine the effects of this combination on the tumor microenvironment. Indeed, previous studies have shown that the inhibition of Src and MEK alone have anti-angiogenic effects in other tumor types [44,45,46]. Given that anti-angiogenic therapies have shown promise in thyroid cancer, combined inhibition of Src and MEK1/2 may be an especially promising therapy by targeting both the tumor and microenvironment [47,48].

## 5. Conclusions

**Figure 6:**
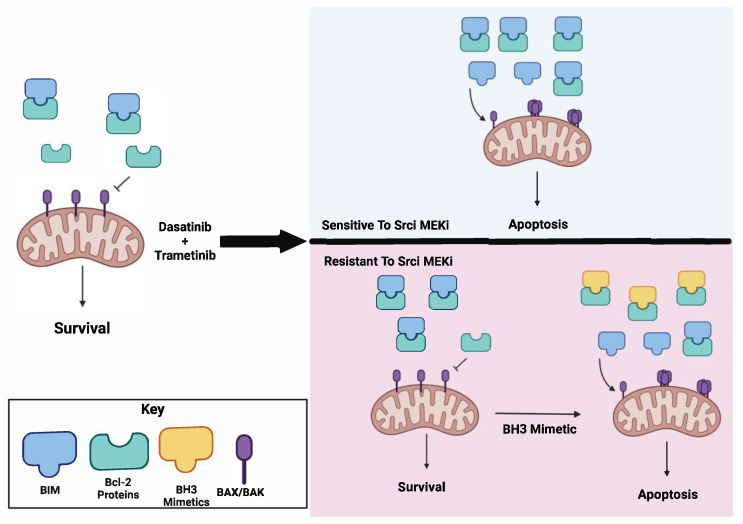
Model summarizing how combined Src and MEK1/2 inhibition induces BIM. Dasatinib and trametinib treatment induces BIM expression prompting thyroid cancer cells to undergo apoptosis in cells that are sensitive. In resistant cells, Src and MEK1/2 inhibition alone does not induce BIM expression high enough to induce apoptosis. The addition of a BH3 mimetic is sufficient to sensitize cells to Src and MEK1/2 inhibition.

To conclude, our findings identified BIM as a mediator of sensitivity to combined Src and MAPK inhibition. We propose, upon treatment with Src and MEK1/2 inhibition, that a sufficient threshold of BIM is induced to initiate apoptosis and render cells sensitive to this combination therapy (Figure 6). Our results further show inhibition of AKT is necessary for sensitivity to combined Src and MEK1/2 inhibition; however, it is predicted to be toxic. As an alternative therapeutic strategy, we demonstrate the addition of the pan BCL-2 BH3 mimetic, ABT-263, is sufficient to induce sensitivity to combined Src and MEK1/2 inhibition.

## Figures and Tables

**Table 1:** Analysis of sensitivity to combined dasatinib and trametinib.

Cell Line	Driver Oncoprotein	IC_50_ nM of Dasatinib + 100 nM Trametinib	Sensitive or Resistant
BCPAP *†	BRAF V600E	0.03	Sensitive
CUTC48	RET/PTC1	0.08	Sensitive
Cal62 *†	KRAS G12R	0.24	Sensitive
C643 *	HRAS G13R	0.28	Sensitive
SW1736 *	BRAF V600E	0.29	Sensitive
K1 *	BRAF V600E PIK3CA E542K W11C	3.08	Sensitive
8505C *†	BRAF V600E	3.99	Sensitive
CUTC5	BRAF V600E	8.83	Sensitive
TPC1	RET/PTC1	11.74	Sensitive
Hth7	NRAS Q61R	15.46	Sensitive
KTC1	BRAF V600E	16.55	Sensitive
OCUT2	BRAF V600E PIK3CA H1047R	18.53	Sensitive
T235	BRAF V600E	21.86	Sensitive
8305C	BRAF V600E	25.77	Sensitive
MDA-T41 *	BRAF V600E	28.00	Sensitive
ACT1	NRAS Q61K	46.53	Sensitive
Hth104	BRAF V600E	46.64	Sensitive
KHM5M	BRAF V600E PIK3CA M10431	62.90	Sensitive
Hth74	None	318.07	Resistant
THJ29T	None	418.47	Resistant
T238 *†	BRAF V600E PIK3CA E542K	722.37	Resistant
CUTC60 *	BRAF V600E	3364.00	Resistant
TCO1 *†	BRAF V600E PIK3CA N1044S	22,637.00	Resistant

IC_50_: concentrations that give 50% growth inhibition. * denotes cell lines used in RPPA. All cell lines were used in gene expression dataset 1. † denotes cell lines used in gene expression dataset 2.

## Data Availability

The data presented in this study will be deposited in NCBI GEO.

## Supplementary Materials

The following supporting information can be downloaded at: https://www.mdpi.com/article/10.3390/cancers15020378/s1, Table S1: Characteristics of the 23 thyroid cell lines used in this study; Table S2: RRID for antibodies used in this study; Table S3: IC_50_ of dasatinib in the presence of 10 nM trametinib; Figure S1: Dasatinib and trametinib treatment enriches apoptosis signature; Figure S2: Knockdown of BIM decreases dasatinib- and trametinib-induced apoptosis; Figure S3: Overexpression of BIM sensitizes resistant cells to growth inhibition and apoptosis induction; Figure S4: Constitutive activation of AKT induces resistance to dasatinib and trametinib; Figure S5: BIM levels remain elevated after removal of dasatinib and trametinib; Figure S6: Original Western blot images.
