# Supplementary material for: BCL2L11 Induction Mediates Sensitivity to Src and MEK1/2 Inhibition in Thyroid Cancer
Source: Cancers (Basel). 2023 Jan 6;15(2):378. doi: 10.3390/cancers15020378 (PMC9856535; doi:10.3390/cancers15020378)

Supplemental Table S1: Characteristics of the 23 thyroid cell lines used in this study

Cell Line	Driver Oncoprotein	Cellosaurus ID	Original Thyroid Tumor Type
CUTC48	RET/PTC1	CVCL_917	Papillary
CUTC5	BRAF V600E	CVCL_W916	Papillary
BCPAP	BRAF V600E	CVCL_0153	Papillary
Cal62	KRAS G12R	CVCL_1112	Anaplastic
TPC1	RET/PTC1	CVCL_6298	Papillary
C643	HRAS G13R	CVCL_5969	Anaplastic
OCUT2	BRAF V600E<br>PIK3CA H1047R	CVCL_9814	Anaplastic
Hth7	NRAS Q61R	CVCL_6289	Anaplastic
K1	BRAF V600E<br>PIK3CA E542K W11C	CVCL_9918	Papillary
KTC1	BRAF V600E	CVCL_6300	Papillary
SW1736	BRAF V600E	CVCL_3883	Anaplastic
T235	BRAF V600E	CVCL_6478	Anaplastic
8505C	BRAF V600E	CVCL_1054	Anaplastic
8305C	BRAF V600E	CVCL_1053	Anaplastic
MDA-T41	BRAF V600E	CVCL_W914	Papillary
Hth104	BRAF V600E	CVCL_A427	Anaplastic
ACT1	NRAS Q61K	CVCL_6291	Anaplastic
KHM5M	BRAF V600E<br>PIK3CA M1043I	CVCL_2975	Anaplastic
T238	BRAF V600E<br>PIK3CA E542K	CVCL_6299	Anaplastic
Hth74	None	CVCL_6288	Anaplastic
THJ29T	None	CVCL_W922	Anaplastic
CUTC60	BRAF V600E	CVCL_VM61	Anaplastic
TCO1	BRAF V600E<br>PIK3CA N1044S	CVCL_M839	Anaplastic

Supplemental Table S2: RRID for Antibodies used in this study

Protein	Species	Supplier	Use	Dilution	WB Blocking	RRID
Phospho-S473 AKT	Rb	CST	IB	1:1000	Li-COR	AB_329825
Total AKT	Ms	CST	IB	1:1000	Li-COR	AB_329827
α-Tubulin	Ms	Calbiochem	IB	1:4000	Li-COR	AB_2617116
β-Actin	Ms	Sigma	IB	1:20000	Li-COR	AB_476744
Phospho-T202/Y204 ERK1/2	Rb	CST	IB	1:1000	Li-COR	AB_2315112
Total ERK1/2	Ms	CST	IB	1:1000	Li-COR	AB_10695739
Phospho-S217/221 MEK1/2	Rb	CST	IB	1:1000	Li-COR	AB_2138017
Total MEK1/2	Ms	CST	IB	1:1000	Li-COR	AB_10695868
Total BIM	Rb	CST	IB	1:500	Li-COR	AB_1030947
Phopsho-Y416 Src	Rb	CST	IB	1:1000	Li-COR	AB_10013641
Total Src	Rb	CST	IB	1:1000	Li-COR	AB_2106059
Phospho-Y861 FAK	Rb	Invitrogen	IB	1:1000	Li-COR	AB_2533703
Total FAK	Ms	BD Biosciences	IB	1:1000	Li-COR	AB_397494

Supplemental Table S3: IC50 of Dasatinib in the Presence of 10 nM Trametinib

Cell Line	IC50 nM of Dasatinib + 10 nM Trametinib
CUTC5	11.6
BCPAP	12.6
Cal62	14.3
CUTC48	18.3
K1	24.6
SW1736	25.4
MDA-T41	28.5
C643	31.3
TPC1	33.9
8505C	42.5
T235	45.2
Hth7	49.6
8305C	54.6
KTC1	62.4
OCUT2	112.5
ACT1	137.3
Hth104	142.8
T238	454.8
KHM5M	521.1
THJ29T	795.3
CUTC60	2447
Hth74	> 4000
TCO1	> 4000

Supplemental Figure S1: (A) Gene set enrichment analysis for hallmark apoptosis signature from Affymetrix data in 23 thyroid cancer cell lines from Table 1 and (B) RNA sequencing data from 3 sensitive (BCPAP, Cal62, 8505C) and 2 resistant (T238 and TCO1) cells. (C) Heatmap of Apoptosis Proteins from MD Anderson Pathway Browser (ID 2991) comparing DMSO treated and combined dasatinib and trametinib treated sensitive cells. * indicates proteins that were statistically significant p = <0.05 (D) BIM expression levels obtained from RPPA in sensitive vs. resistant cells treated with DMSO, 50 nM dasatinib, 100 nM trametinib, or the combination 2-way ANOVA * p = 0.02

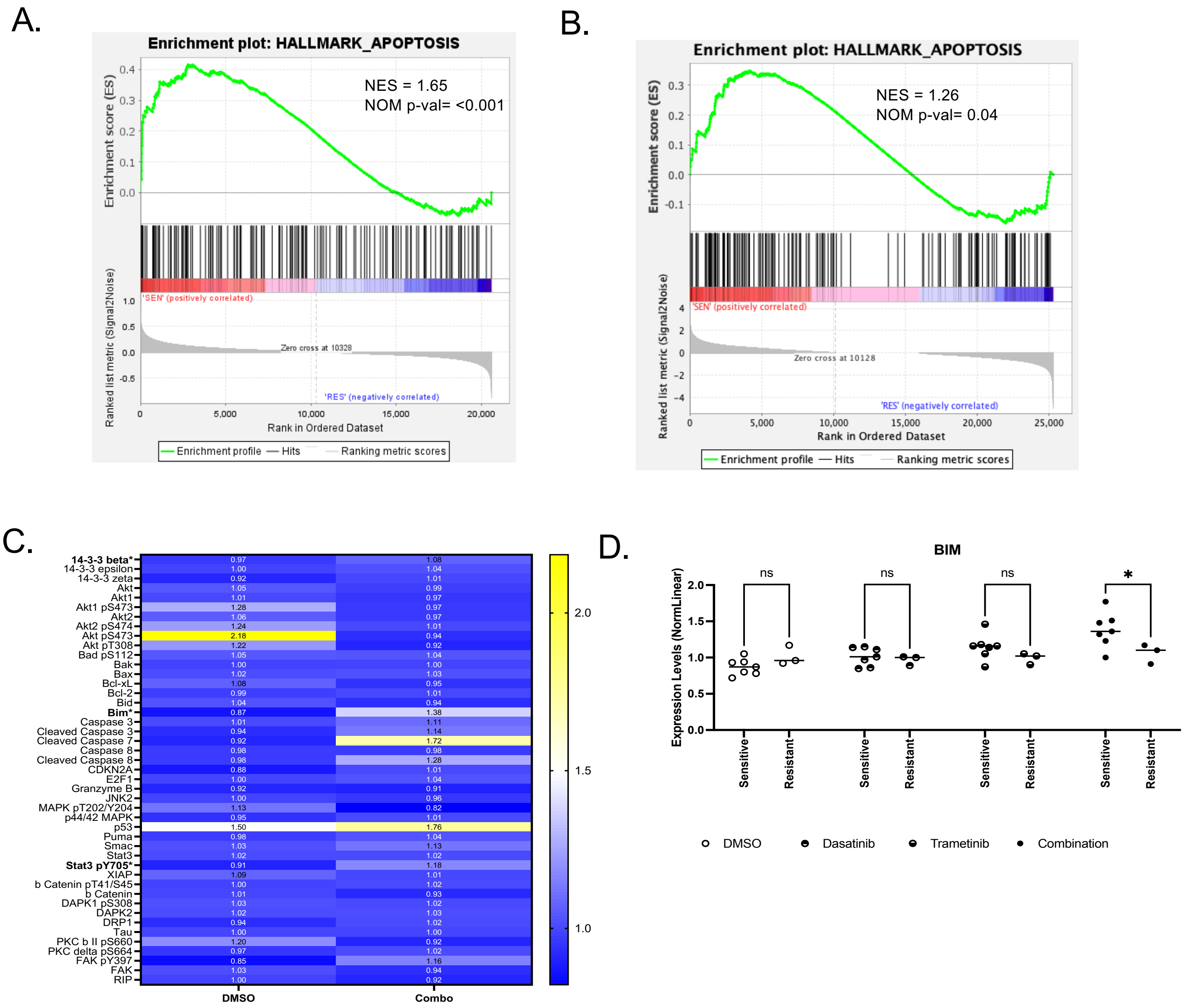

Supplemental Figure S1 continued: (E) Immunoblot analysis of 8505C, T238, CUTC60 cells were treated with indicated concentrations of dasatinib, trametinib, or the combination for 24 hrs. and analyzed by Western blot for expression of indicated antibodies. Alpha tubulin was used as a loading control. Numbers below represent densitometric analysis normalized to loading control followed by DMSO treated cells.

E.

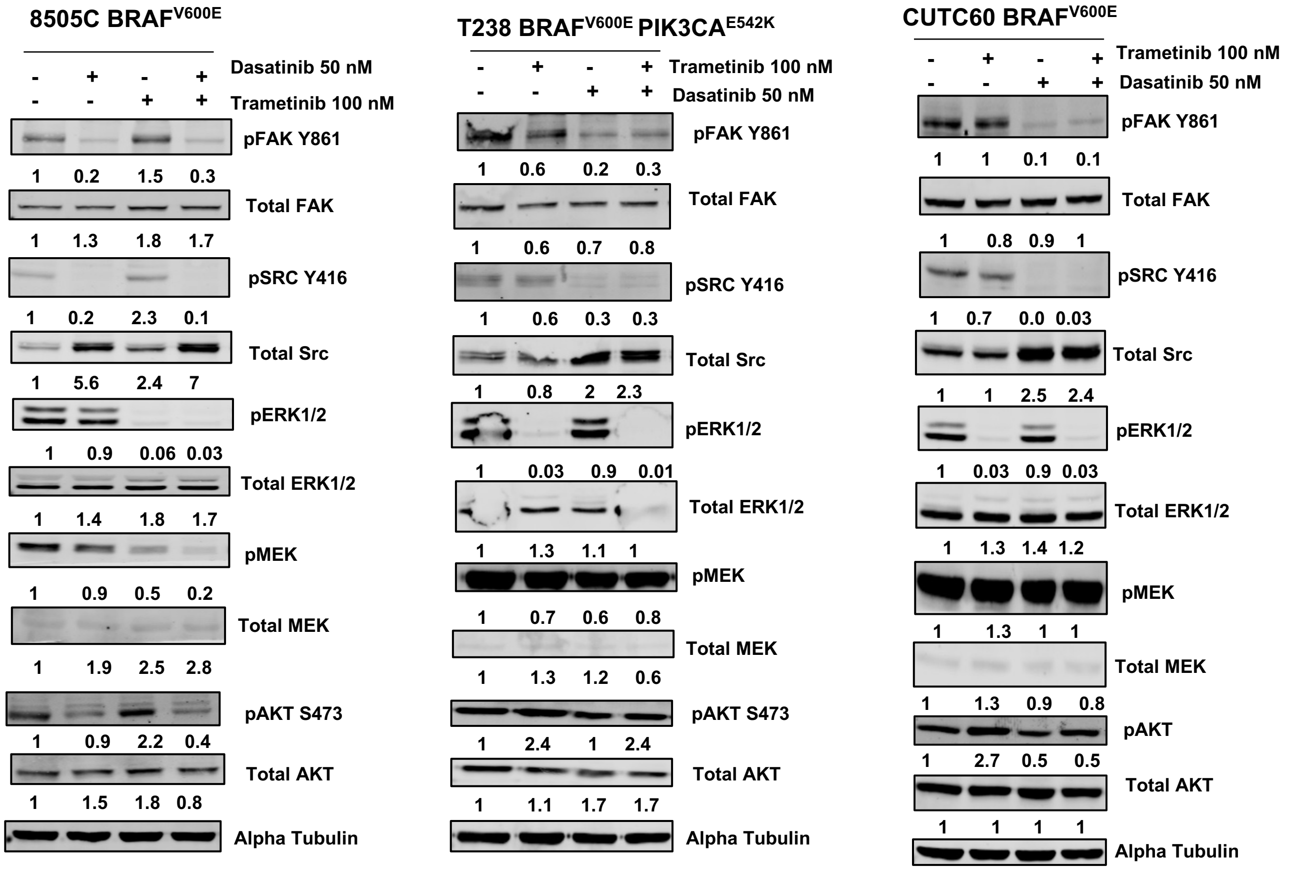

Supplemental Figure S2: Annexin V/PI Flow plots of 8505C cells reverse transfected with nontargeting or BIM siRNA and treated with DMSO or combined 50 nM dasatinib and 100 nM trametinib. Cell death percent was calculated by adding apoptosis dead and early apoptosis quadrants. 2-way ANOVA was performed **p = 0.002 *p = 0.02

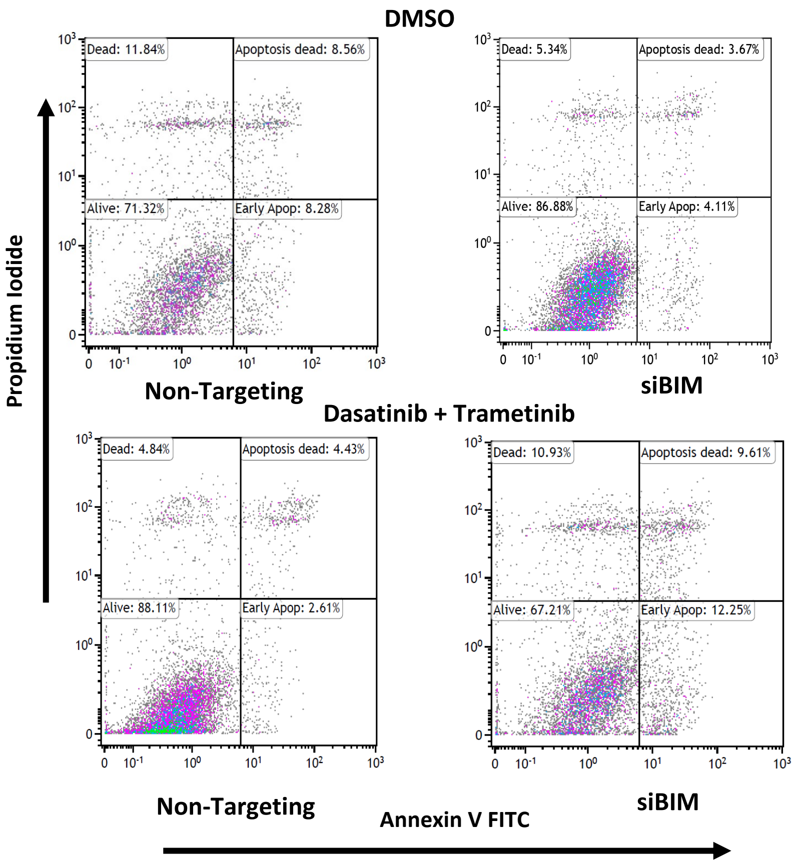

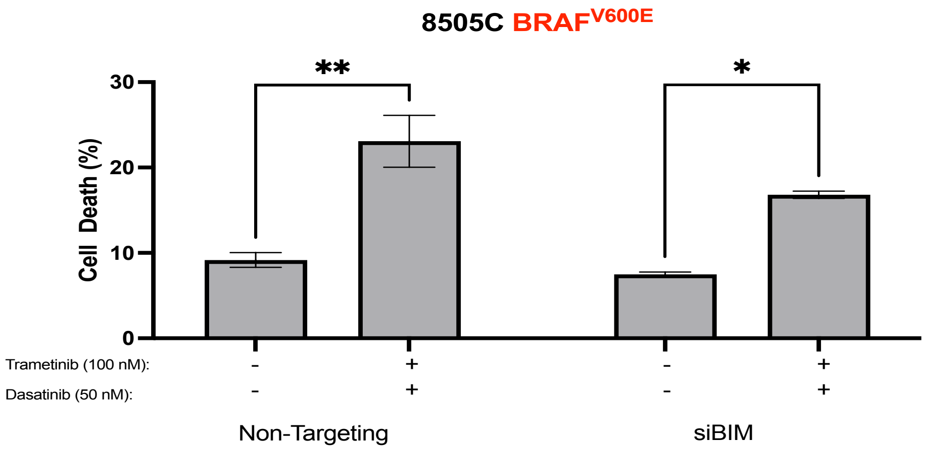

Supplemental Figure S3: (A) CellTiter-Glo of T238 and CUTC60 parental cells, empty vector, cells, and BIM overexpressing cells treated with and without 1 ng/mL of doxycycline and indicated doses of dasatinib in the presence of 100 nM trametinib for 72 hrs. Data was normalized to DMSO-treated control set to 100%. Dashed line represents 50% viability. Results shown are mean ± SEM (B) Cleaved caspase 3/7 activity in T238 and CUTC60 cells expressing empty vector or BIM plasmid and treated with 1 ng/mL of doxycycline and DMSO, 50 nM dasatinib, 100 nM trametinib, or the combination was measured over 72 hours and graphed as area under the curve. 2-Way ANOVA *p < 0.05 **p < 0.01 ***p < 0.0005 ****p < 0.0001

A.

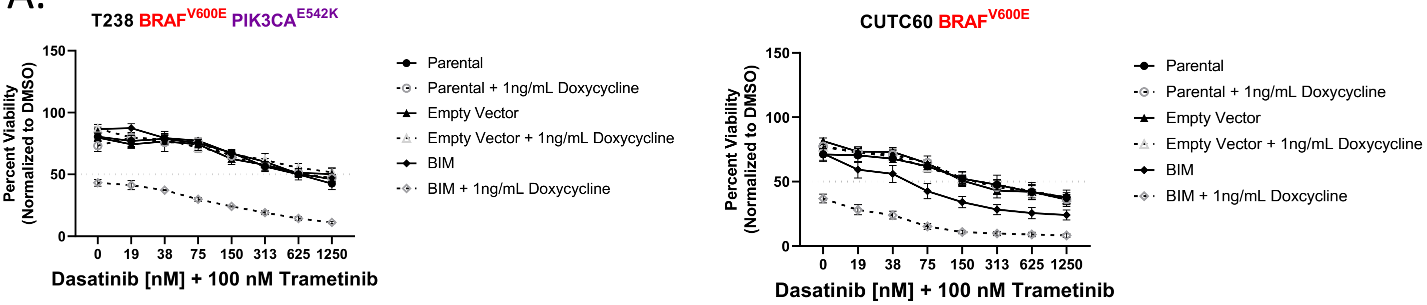

B.

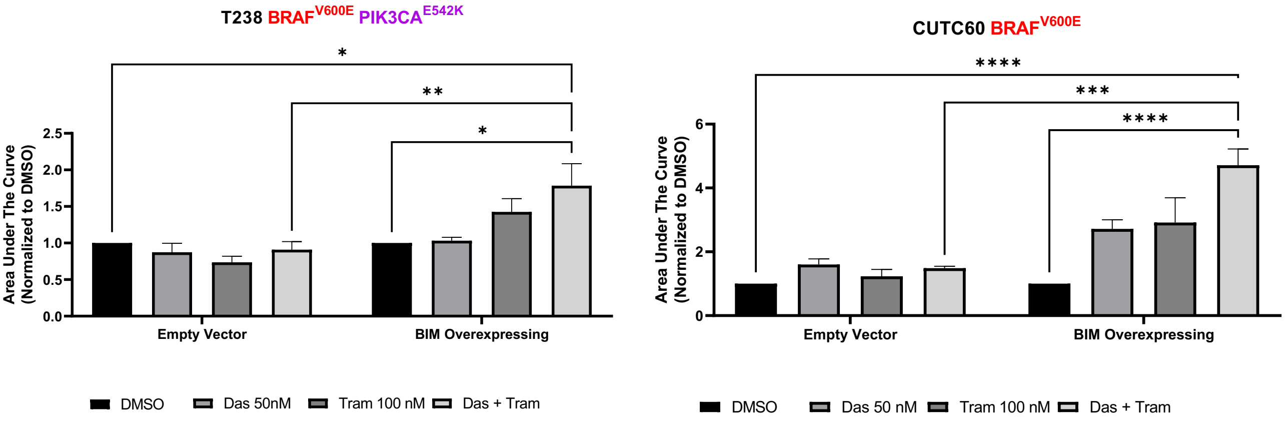

Supplemental Figure S4: CellTiter Glo of 8505C Parental, Empty Vector, and Myr AKT expressing cells treated with indicated doses of dasatinib in the presence of 9, 1, 10, or 100 nM trametinib for 72 hrs. Data was normalized to DMSO-treated control set to 100%. Dashed line represents 50% viability. Results shown are mean ± SEM

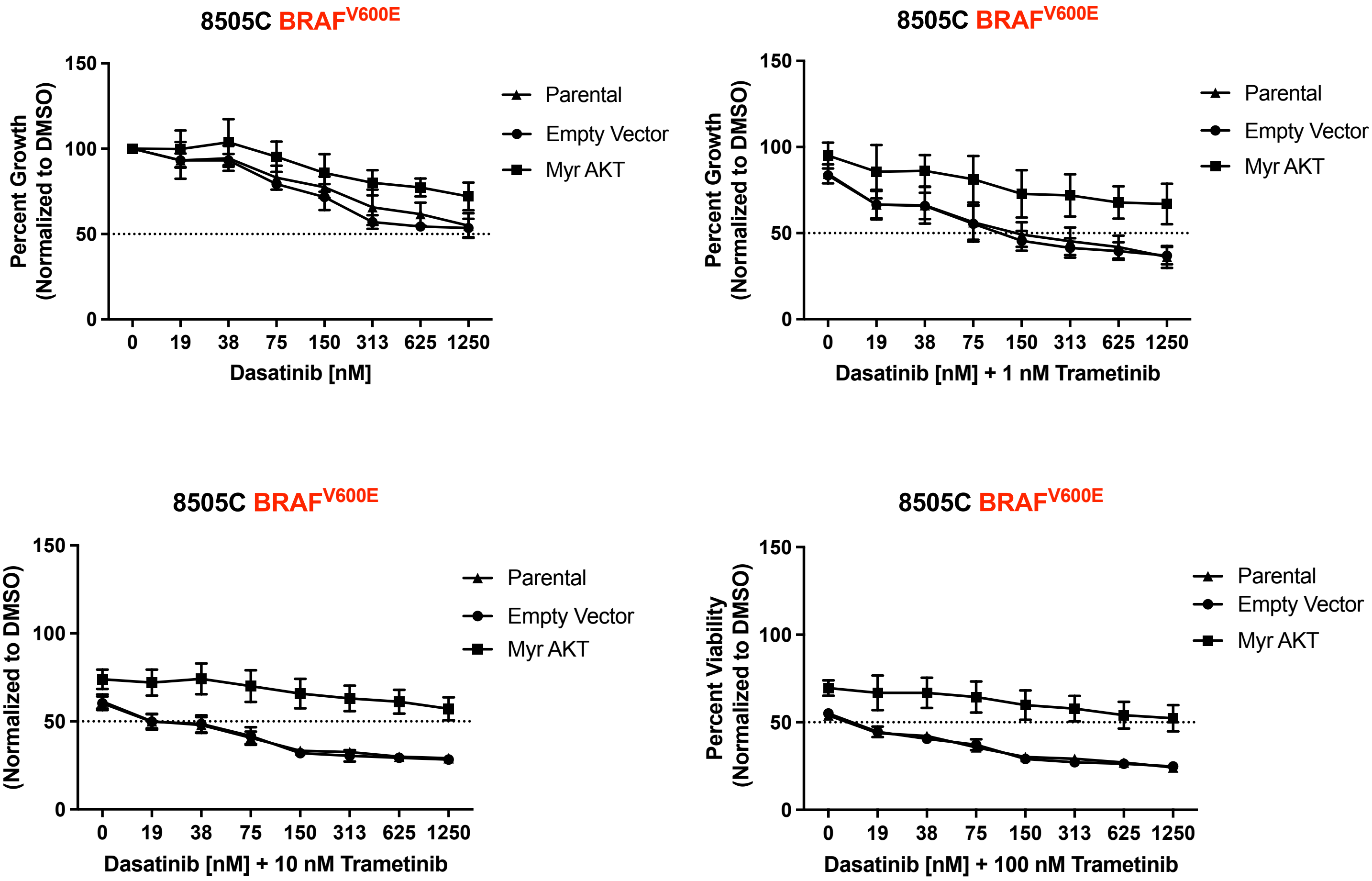

Supplemental Figure S5: Immunoblot analysis of 8505C and T238 cells treated with DMSO or 50 nM dasatinib and 100 nM trametinib for 24 hours. After 24 hours drug was removed, and lysates were harvested at the indicated time points and analyzed by Western blot for expression of BIM. Alpha tubulin was used as a loading control Numbers below represent densitometric analysis normalized to loading control followed by DMSO treated cells.

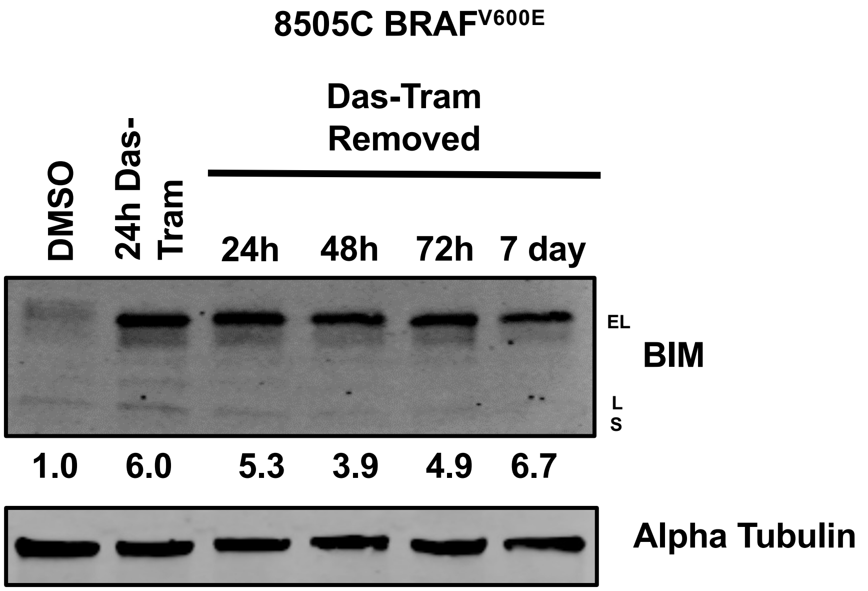

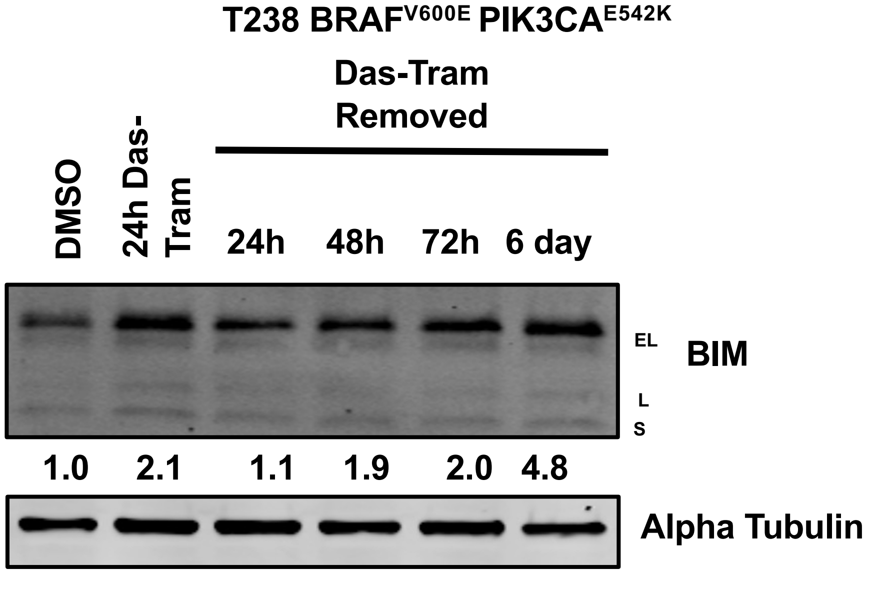

Supplemental Figure S6: Original Western Blots

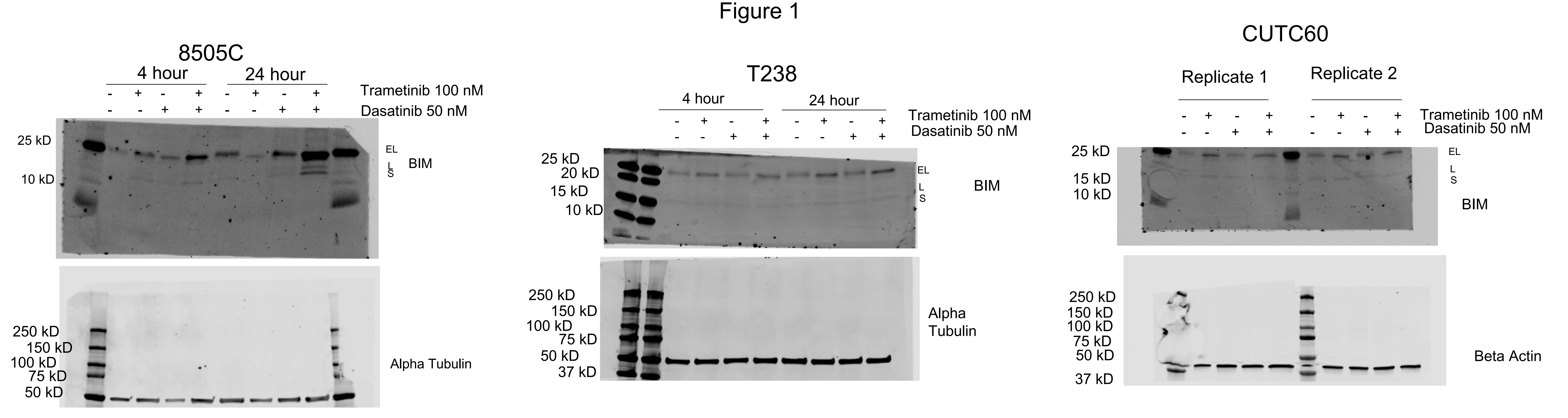

Supplemental Figure S6: Original Western Blots

Figure 2

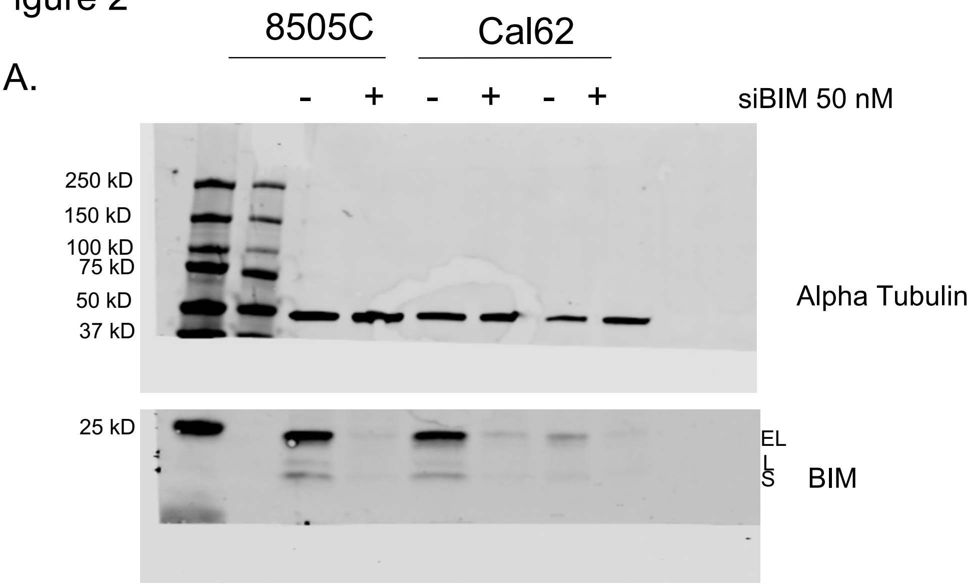

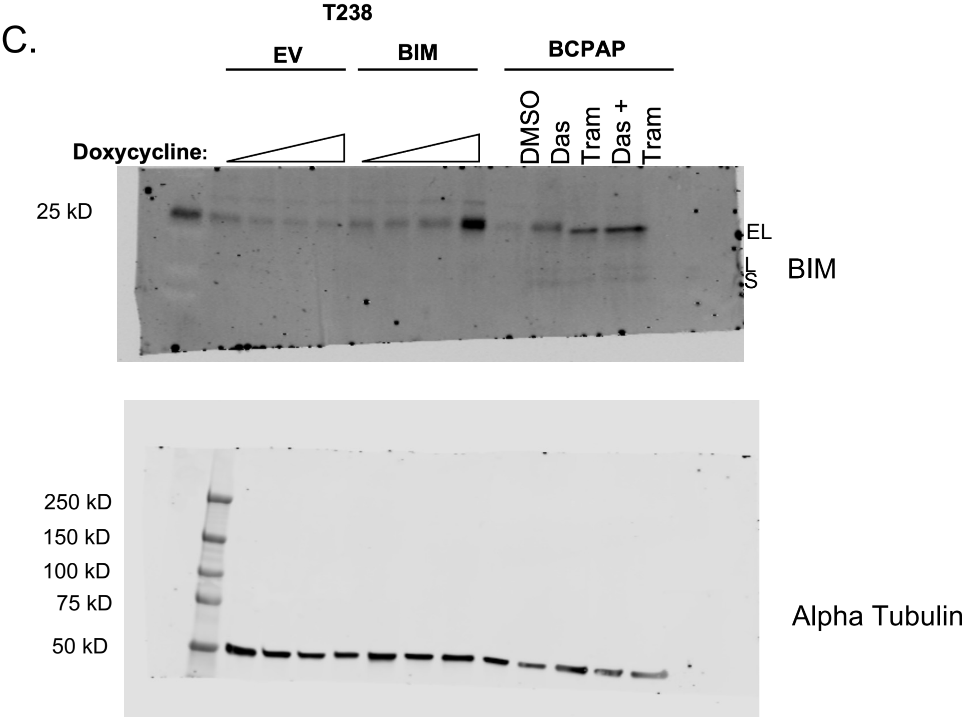

Supplemental Figure S6: Original Western Blots

Figure 3 8505C $BRAF^{V600E}$

A.

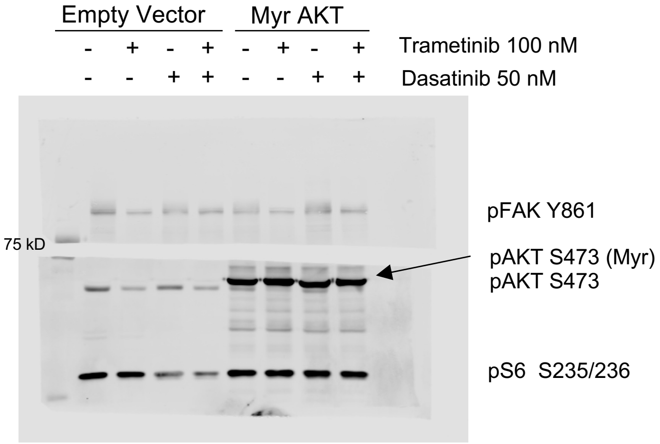

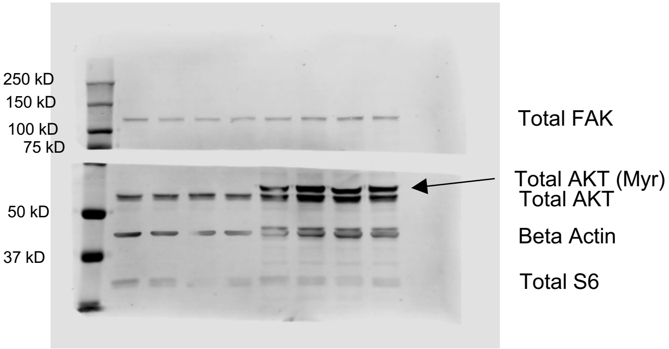

D.

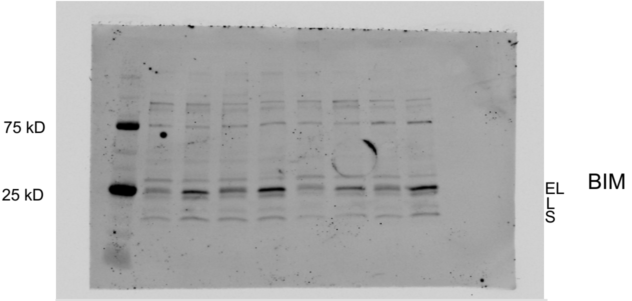

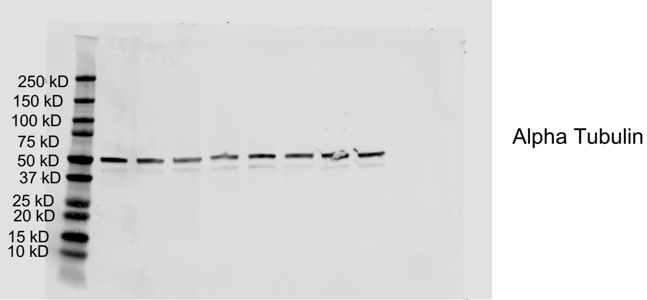

Supplemental Figure S6: Original Western Blots

Figure S1

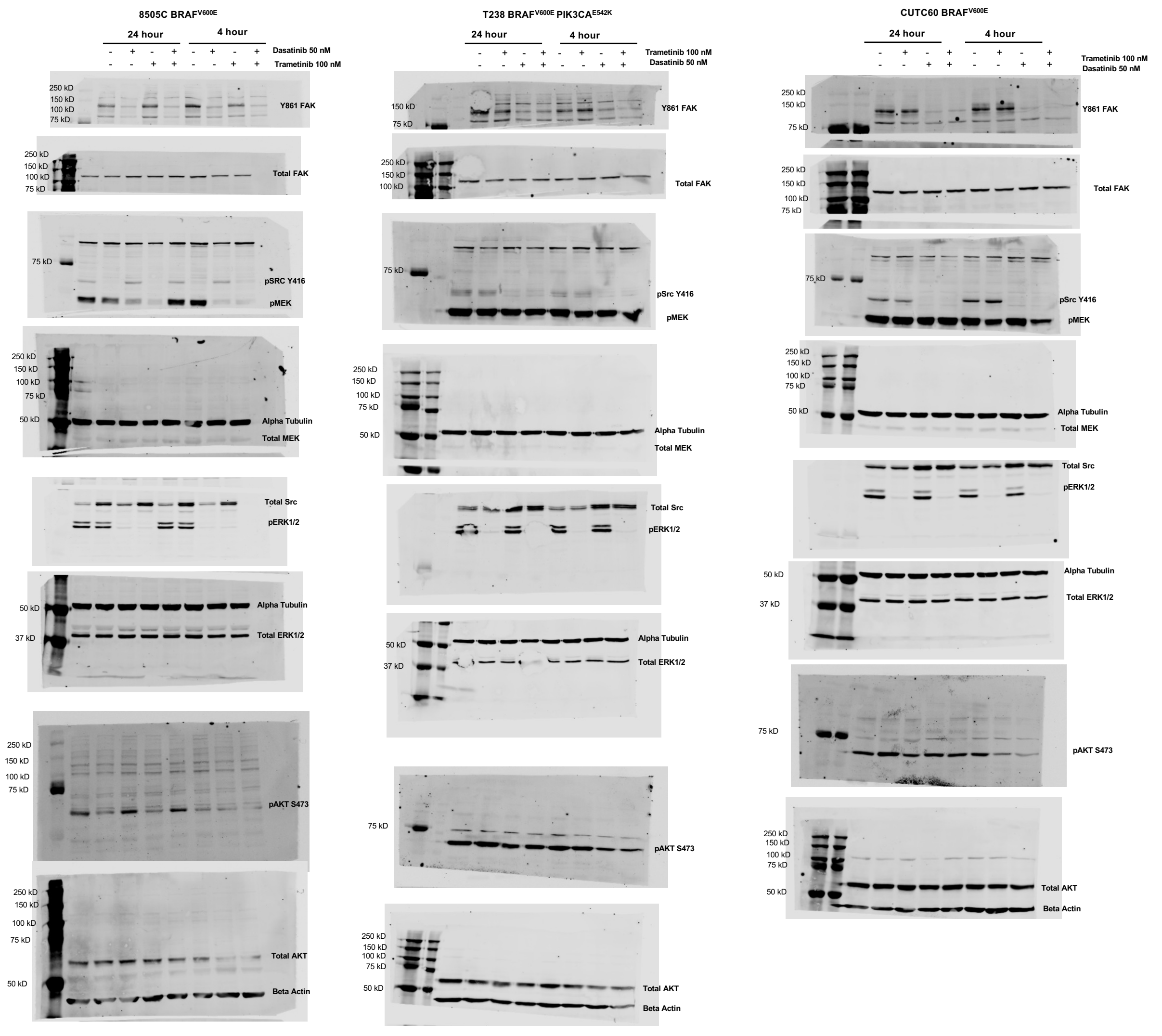

Supplemental Figure S6: Original Western Blots

Figure S5

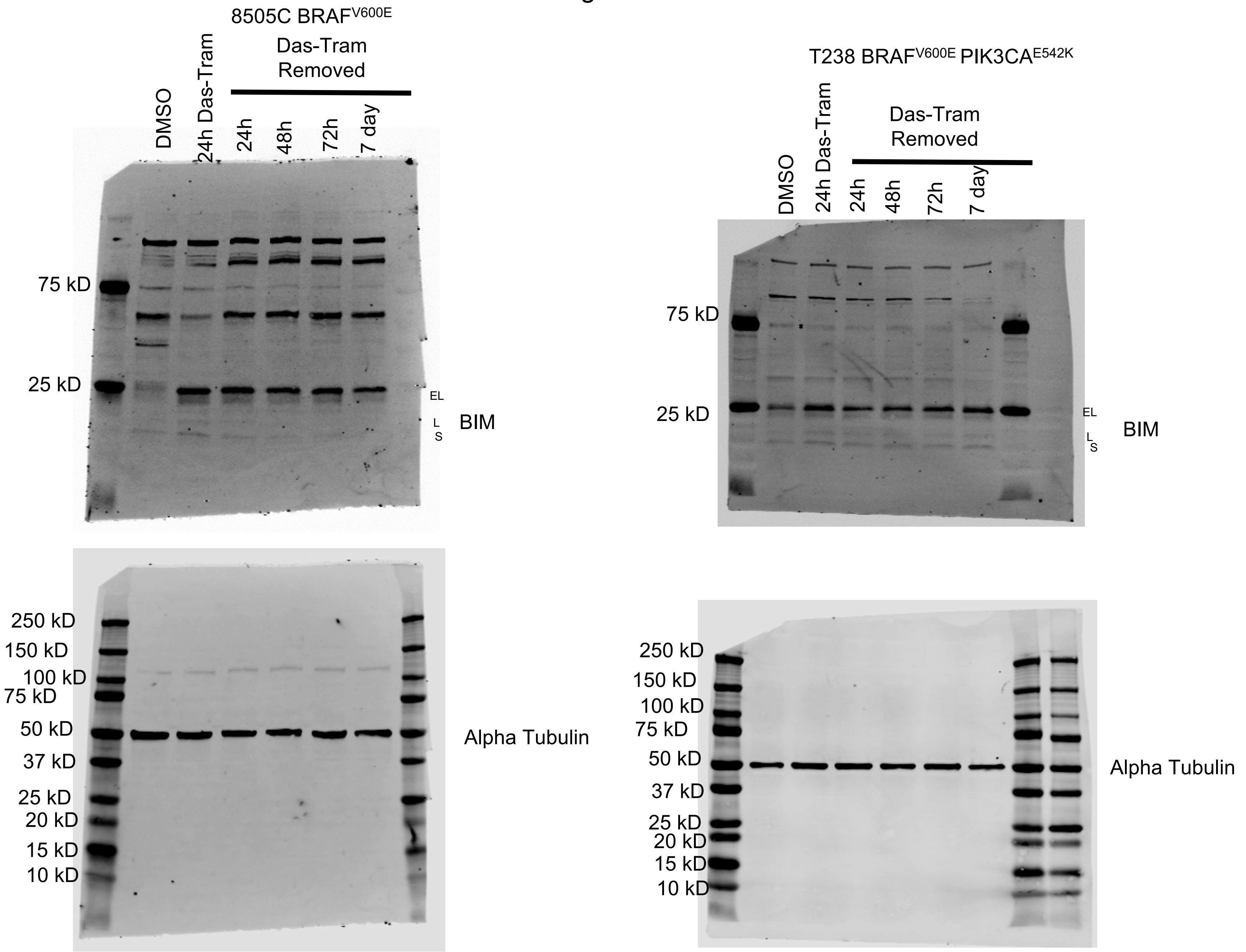

Supplement: Supplementary file 1 [file cancers-15-00378-s001.zip › cancers-2116871-supplementary.pdf]
